# Image motion with color contrast suffices to elicit an optokinetic reflex in *Xenopus laevis* tadpoles

**DOI:** 10.1038/s41598-021-87835-2

**Published:** 2021-04-19

**Authors:** Alexander G. Knorr, Céline M. Gravot, Stefan Glasauer, Hans Straka

**Affiliations:** 1 Computational Neuroscience, Brandenburg University of Technology Cottbus-Senftenberg, Universitätsplatz 1, 01968 Senftenberg, Germany; 2 Department Biology II, Ludwig-Maximilians-University Munich, Großhaderner Str. 2, 82152 Planegg, Germany; 3 Graduate School of Systemic Neurosciences, Ludwig-Maximilians-University Munich, Großhaderner Str. 2, 82152 Planegg, Germany

**Keywords:** Colour vision, Motion detection, Reflexes

## Abstract

The optokinetic reflex is a closed-loop gaze-stabilizing ocular motor reaction that minimizes residual retinal image slip during vestibulo-ocular reflexes. In experimental isolation, the reflex is usually activated by motion of an achromatic large-field visual background with strong influence of radiance contrast on visual motion estimation and behavioral performance. The presence of color in natural environments, however, suggests that chromatic cues of visual scenes provide additional parameters for image motion detection. Here, we employed *Xenopus laevis* tadpoles to study the influence of color cues on the performance of the optokinetic reflex and multi-unit optic nerve discharge during motion of a large-field visual scene. Even though the amplitude of the optokinetic reflex decreases with smaller radiance contrast, considerable residual eye movements persist at the ‘point of equiluminance’ of the colored stimuli. Given the color motion preferences of individual optic nerve fibers, the underlying computation potentially originates in retinal circuits. Differential retinal ganglion cell projections and associated ocular motor signal transformation might further reinforce the color dependency in conceptual correspondence with head/body optomotor signaling. Optokinetic reflex performance under natural light conditions is accordingly influenced by radiance contrast as well as by the color composition of the moving visual scene.

## Introduction

Gaze-stabilization largely depends on the concerted action of synergistic vestibulo-ocular (VOR) and optokinetic reflexes (OKR)^1^. The latter reflex is elicited by large-field visual field motion, operates under closed-loop conditions and ensures minimization of the residual image slip that remains uncompensated by the open-loop VOR^1–3^. In all vertebrates, this reflex is relayed by a distinct retinal circuitry, ganglion cell pathway and central nucleus in the pretectum that constitutes the accessory optic system and mediates neuronal signals both directly and indirectly to specific sets of extraocular motoneurons for eye movements^2,4^. Based on the circuitry, this reflex differs distinctly from optomotor responses for orienting and stabilizing head/body movements, which are mediated by the regular retinal ganglion cell (RGC) pathway, pretectal, but mostly tectal circuits, tecto-spinal and tecto-bulbo-spinal pathways as elaborated in detail in zebrafish^5,6^.

Visual image motion-driven ocular motor responses have been shown to rely on an internal estimate of the velocity of the visual scenery^7,8^ and are strongly influenced by image characteristics of the motion stimulus^5,9,10^. During experimentation, optokinetic reflexes have been classically elicited by achromatic image motion that purely depended on contrast differences. Natural environments, however, typically contain multiple qualities of visual information parameters that potentially can be used to detect and estimate visual field motion^11^. Typically, a visual scene is structured by luminance (although the term luminance strictly is a SI unit, it is used here to denote the subjective light intensity) and color cues, both of which are able to provide information about self- and object motion^5,10,12^. While visual environments are generally well defined by local changes in light intensity, adjacent visual field areas might have the same brightness, but a different color. Effective image motion detection under such conditions therefore requires that neuronal processing of visual motion also includes information about color. In fact, early studies in larval and adult frogs have shown that at least optomotor head/body orienting responses can be triggered by the motion of colored visual scenes^13,14^, further elaborated in some detail more recently in zebrafish larvae^5^. However, the impact of color contrast on the performance of optokinetic ocular motor responses is still largely unknown.

The precise role of color information in motion perception has been a matter of long debate^15^. Early studies purported that color and motion are encoded and processed in strict separation based on neurophysiological and lesion studies^16^. In contrast, motion detection of two equiluminant color patterns in human psychophysical experiments^17^ provided evidence that color contributes to motion perception^18,19^. This latter notion is supported by several studies in primates that all appear to indicate that color information contributes to visual motion detection^20^. In particular, color cues can induce ocular following responses, suggesting that color and luminance information are jointly processed during the generation of visuo-ocular motor responses^20^. Processing of chromatic motion cues, however, was so far associated with higher-level cortical mechanisms^21–23^. However, a differential contribution of color cues to the OKR has been demonstrated in larval Axolotl and *Xenopus*^12^ and thus in the absence of a cortex. The mediation of the OKR by retinal and brainstem circuits suggests that neuronal processing of color contrast is a conserved feature and might play a fundamental role in motion estimation and gaze-stabilization throughout vertebrates.

Here, we employed semi-intact preparations of *Xenopus laevis* tadpoles to test the hypothesis that luminance and color information of a moving visual scene are concurrently exploited and integrated at the level of the brainstem into visuo-ocular motor commands for the OKR. With our study we follow an approach previously introduced by Knorr et al.^12^, in which the involved biophysical and physiological processes are treated as a ‘black box’. As such, we were interested in how the observed behavioral responses are related to the experimental sensory stimuli, however, without making claims about internal representation, perception, color discrimination under static conditions, or intermediate processing mechanisms. Measurements of ocular motor performance under different conditions confirmed a non-zero ocular motor response at iso-luminance and recordings of RGC activity revealed distinct preferences for individual colors of the visual motion stimulus. Part of these results found entry into the thesis of CMG^24^.

## Material and methods

### Animals

Experiments were performed in vitro on semi-intact preparations of *Xenopus laevis* tadpoles (*n* = 46) and complied with the "Principles of animal care", publication No. 86-23, revised 1985 of the National Institute of Health. All experiments were carried out in accordance with ARRIVE guidelines and regulations. Permission for these experiments was granted by the ethics committee for animal experimentation of the legally responsible governmental institution (Regierung von Oberbayern) under the license code 55.2-1-54-2532.3-59-12. In addition, all experimental methods were performed in accordance with the relevant guidelines and regulations of the Ludwig-Maximilians-University Munich. *Xenopus* larvae of either sex at stage 51–54^25^ were obtained from the in-house animal breeding facility at the Biocenter-Martinsried of the Ludwig-Maximilians-University Munich. Animals were maintained in tanks with non-chlorinated water (17–18 °C) at a 12/12 light/dark cycle prior to experimentation.

### Experimental approach

Semi-intact preparations were obtained according to the procedure reported previously^10,26^. In brief, tadpoles were anesthetized in 0.05% 3-aminobenzoic acid ethyl ester (MS-222; Pharmaq) in frog Ringer solution (in mM: 75 NaCl, 25 NaHCO_3_, 2 CaCl_2_, 2 KCl, 0.5 MgCl_2_ and 11 glucose, pH 7.4) and decapitated. For experiments that employed eye motion recordings, the skin covering the dorsal head was removed, the soft skull tissue opened and the forebrain disconnected. This surgical procedure anatomically and functionally preserved the remaining CNS with the eyes and associated optic nerve, extraocular motor innervation and eye muscles. Such preparations allowed prolonged experimentation and in vivo-like activation of the OKR by horizontal large-field visual image motion under defined in-vitro conditions^10,12^. For electrophysiological recordings of RGC axons in these preparations, the optic nerve of the right eye was cleaned from surrounding connective tissue and transected before entering the optic chiasm. All extraocular muscles of this eye were transected at their proximal insertion site to immobilize the eye in its natural position within the head. After the surgery, all preparations were allowed to recover from the surgical intervention for three hours^27^.

### Eye motion capture and optic nerve recordings

Semi-intact preparations were mechanically secured with insect pins to the Sylgard floor of a Petri dish (5 cm in diameter). As described earlier^12^, the chamber, which was constantly perfused with oxygenated frog Ringer solution at a rate of 3.0–5.0 ml/min, was placed in the center of an open cylindrical screen with a height of 5 cm and a diameter of 8 cm, encompassing a horizontal visual field of 275°. Three digital light processing (DLP) video projectors (Aiptek V60), installed at 90° angles to each other projected visual motion stimuli onto the screen^12,28^ at a refresh rate of 60 Hz. For eye motion recordings, a CCD camera (Grasshopper 0.3 MP Mono FireWire 1394b, PointGrey, Vancouver, BC, Canada), mounted 20 cm above the center of the recording chamber, permitted on-line tracking of horizontal eye movements by custom-written software^29^. The position of both eyes was digitized at a sampling rate of 50 Hz and recorded along with the visual motion stimulus (Spike2 version 7.04, Cambridge Electronic Design Ltd., Cambridge, United Kingdom). The chamber was illuminated from above using an 840 nm infrared light source. An infrared long-pass filter inside the camera ensured selective transmission of the respective wavelengths and a high contrast to outline the eyeballs for motion tracking and online analysis of induced eye movements.

Electrophysiological recordings of multi-unit optic nerve spike activity were performed under the same experimental conditions as described previously^10^. In brief, the spike discharge of retinal ganglion cells was recorded extracellularly (EXT 10-2F, npi Electronics, Tamm, Germany) with glass microelectrodes that were filled with Ringer solution^10^. Electrodes were produced with a horizontal puller (P-87 Brown/Flaming, Sutter Instruments Company, Novato, CA, USA) and the tips were broken and individually adjusted to fit the diameter of the transected optic nerve^10^. The spike discharge was digitized at a sampling rate of 28.6 kHz (CED Micro1401-3, Cambridge Electronic Design Ltd., Cambridge, UK) together with the visual motion stimulus. The discharge was recorded by a data acquisition program (Spike2 version 7.04, Cambridge Electronic Design Ltd., Cambridge, UK) and stored for off-line spike-sorting to extract single-unit activity (see below).

### Stimulus paradigms

**Figure 1:**
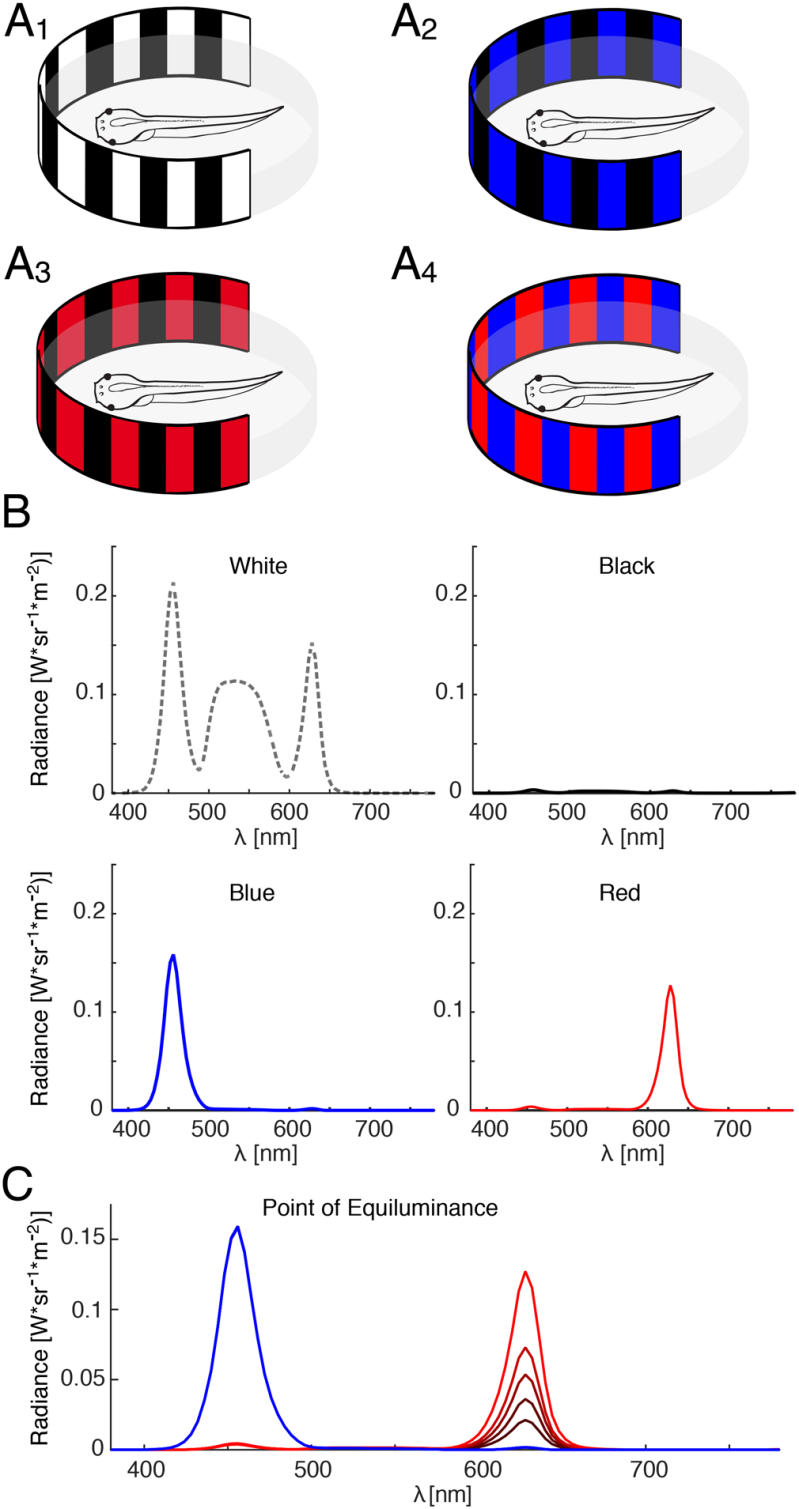
Color patterns and wavelength spectrum of large-field visual motion stimuli. **(A)** Schematics of color motion stimuli with black–white **(A**_**1**_**)**, black–blue **(A**_**2**_**)**, black–red **(A**_**3**_**)** and red–blue **(A**_**4**_**)** vertical stripes that were projected onto a cylindrical screen around the centered semi-intact preparation of a *Xenopus* tadpole. **(B)** Spectra of white, black, blue and red light emitted by the projectors at maximal intensity. **(C)** Graph depicting the method to identify the point of equiluminance; note that the intensity of the blue stripes was held constant, whereas the intensity of the red stripes was gradually altered. Figure assembled with Affinity Designer (version 1.8.3).

A reliable estimate of color motion perception is provided by the performance of visuo-ocular motor responses evoked by moving chromatic and radiance contrast stimuli^12^. This method depends on the brightness ratio of two colors at which the response is minimal^30,31^. To identify this ratio, animals were presented with horizontal visual image motion stimuli using a vertically striped pattern with alternating red and blue vertical stripes. With our digital projection system, the red and blue color provided the maximal possible separation of the wavelength spectrum (Fig. 1B). The intensity of the red stripes was varied systematically, leading to variations in the OKR response due to resulting differences in radiance contrast. At the point of equiluminance (POE), the respective brightness of the optic scene appears to be homogeneous to the animal, such that the visual image is only structured by the color of the scenery. At the POE, a visuo-motor response can either be absent, indicative of a color-blind motion perception system or show a residual response, indicating that color information provides motion cues^12^. Here, we exploited the robust OKR of larval *Xenopus*^10^ that is elicited by horizontal motion of a large-field visual scene (Fig. 1A1-4) with a rectangular velocity profile of ± 10°/s and a frequency of 0.2 Hz.

### Point of equiluminance

The POE was determined by presenting a visual stimulus that consisted of alternating red and blue vertical chromatic stripes (Fig. 1A4). The radiance of the red stripes was varied over a range that extended from 0.29 to 3.18 W*sr^−1^ m^−2^, while the intensity of the blue stripes was maintained at a constant value of 4.66 W*sr^−1^ m^−2^. As a control condition to determine the amplitude of spontaneous eye oscillations or measurement noise, the amplitude of eye movements at the stimulus frequency, while presenting a uniformly lit grey screen, served as reference level for comparison of the minimum OKR amplitude (Grey condition, radiance 3.21 W*sr^−1^ m^−2^). Importantly, the POE was determined individually for each animal as intensity value of the red stripes at which the OKR response was minimal. The mean POE was constructed by averaging the separately obtained values of each animal.

### Interaction of color and motion at high light intensities

Three visual motion stimuli were presented at high intensity levels in random order to assess the interaction of color and radiance motion at high contrast levels. Stripes were white (18.7 W*sr^−1^ m^−2^), red (3.39 W*sr^−1^ m^−2^), blue (4.66 W*sr^−1^ m^−2^) or black (0.187 W*sr^−1^ m^−2^) and were presented in alternating combinations of white/black, red/black, blue/black and red/blue stripes (Fig. 1A1-4).

### Retinal ganglion cell discharge at high light intensities

The encoding of colored motion stimuli at high intensities at the neuronal level was determined by recordings of the optic nerve discharge during horizontal motion of a large-field visual scene. The motion stimulus had a sinusoidal profile with a peak velocity of ± 10°/s and a frequency of 0.125 Hz causing similar positional stimulus oscillations as those used to evoke eye movements.

### Retinal ganglion cell discharge near the POE

To determine whether the discharge of retinal ganglion cells is exclusively driven by radiance contrast, or whether particular units also respond to pure color contrast, the spike activity was determined in response to red/blue stripes at three different red color intensities close to the POE (1.14, 1.19 and 1.25 W*sr^−1^ m^−2^), while the intensity of the blue stripes was kept constant at 4.66 W*sr^−1^ m^−2^.

### Spectral distribution of color stimuli

Individual red and blue colors were generated using the red and blue channels of the image projectors; white stripes were generated using all three color channels (for spectra see Fig. 1B,C). This procedure was chosen for the following reason: if red–black or blue–black stimuli would cause larger responses than white–black stimuli, then the neuronal transformation of RGC signals into optokinetic eye movements must depend on specific chromatic contrasts rather than pure radiance/luminance responses. Thus, if, for example, red-responsive RGCs would be exclusively responsible for eliciting the OKR, then it can be expected that the response to white–black stripes is equal or even stronger than red–black stripes, because red-responsive RGCs would be activated even better with white light (see spectral composition in Fig. 1B). Spectra and radiance values were measured using a spectrometer (PhotoResearch SpectraScan PR655).

### Data analysis

To obtain a robust measure of the strength of the optokinetic response, the OKR magnitude was computed by fitting a triangular profile to the recorded eye position trace and evaluating the amplitude of the fit^12^. To account for the fact that the “actual” value of the POE might lie between the sampled responses at different intensities, the resulting intensity-amplitude curve was fitted with a function for the normalized OKR amplitude *A* defined by:

$$A=m*\frac{\left|{b}_{Red}-{b}_{Blue} \right|}{\left|{b}_{Red}-{b}_{Blue}\right|+c}+{A}_{CR},$$

with the subjective brightness for red or blue:

$${b}_{Red,Blue}=\mathrm{log}\left({L}_{Red, Blue}+1\right)*{c}_{Red,Blue}$$

L_Red_ and L_Blue_ represent the radiance values for red and blue and c_Red_ and c_Blue_ are the relative sensitivities to red and blue, respectively. The parameters *m* and *c* were required to model the individual sensitivity of each preparation’s optokinetic response to changes in contrast. This model was able to fit the observed data, particularly near the POE. The POE was identified as the radiance value of the red stripes at which the fitted function became minimal (A_CR_) individually for each preparation. The chromatic response (CR) component of the optokinetic eye movement was then defined as the OKR amplitude at this point.

### Spike sorting

To determine the response properties of individual retinal ganglion cells, spike sorting was performed in MATLAB (R2017a) on multi-unit optic nerve spike discharge in selected experiments, where individual units were identifiable. For each preparation, all trials were pooled following detection of the action potentials by a non-linear energy operator, the Teager-Kaiser energy operator^32^, with an optimized threshold^33^. Artifacts were identified as all segments of the dataset in which the electrical signal was larger than 1000 mV and were subsequently removed by replacing the respective values with zero. Spikes were then extracted in a 7 ms window around their respective peaks (200 samples at a sampling rate of 28.6 kHz). The collected spike shapes were subjected to singular value decomposition. The three largest singular values were then used for spike sorting. The values were clustered using k-means clustering (MATLAB 2017a). The number of clusters was determined by visual inspection of the 3D scatter plot of singular values to ensure that data were clustered into non-overlapping regions. Spike clustering was very clear in some cases and somewhat less clear in others; only clusters that could be clearly distinguished were taken into account for an evaluation of the respective spike activity of usually up to four units. Firing rate magnitude of individual RGCs was determined as the total count of action potentials during each trial. Since all trials had the same duration and velocity profile, this gave a robust estimate of single-unit activity during stimulation with differently colored stripe combinations.

### Statistical procedures

The significance value was chosen as *α* = 0.05 for all statistical tests. The response amplitudes of the OKR at the POE were compared to the reference values obtained from the *grey* condition using a two-sample t-test. The OKR amplitudes in response to white/black, blue/black, red/black and red/blue vertical stripe motion were compared using a repeated measures ANOVA. As described earlier^12^, post-hoc-tests were performed using paired t-tests between all conditions and the Bonferroni method to compensate for multiple comparisons. To reveal a possible linear interaction between color and radiance motion information, the correlation between the chromatic component of the OKR and the differences between the responses to red/blue, red/black, blue/black and white/black stimuli was computed. To account for potential mathematical coupling, statistical significance of correlations was assessed by randomly re-sampling data points to estimate the distribution of expected correlation coefficients^12^. To determine the response magnitude of individual optic nerve fibers to colored compared to pure radiance stimuli, color preference (g_Red, Blue_), determined as relative increase or decrease of activity in response to colored versus white stimuli was measured by computing the log of the ratio of spike counts (SC) in either red or blue color condition over the spike count in white condition: ${g}_{Red, Blue}=\mathrm{log}\left(\frac{{SC}_{Red,Blue}}{{SC}_{White}}\right).$

### Presentation

Schemes, data and analysis plots were generated with MATLAB (R2017a) and assembled in Affinity Designer (version 1.8.3, Serif Europe, UK).

### Ethics statement

All experiments were carried out in accordance with ARRIVE guidelines and regulations. Permission for these experiments was granted by the ethics committee for animal experimentation of the legally responsible governmental institution (Regierung von Oberbayern) under the license code 55.2–1-54–2532.3–59-12. In addition, all experimental methods were performed in accordance with the relevant guidelines and regulations of the Ludwig-Maximilians-University Munich.

## Results

### Determination of the point of equiluminance

**Figure 2:**
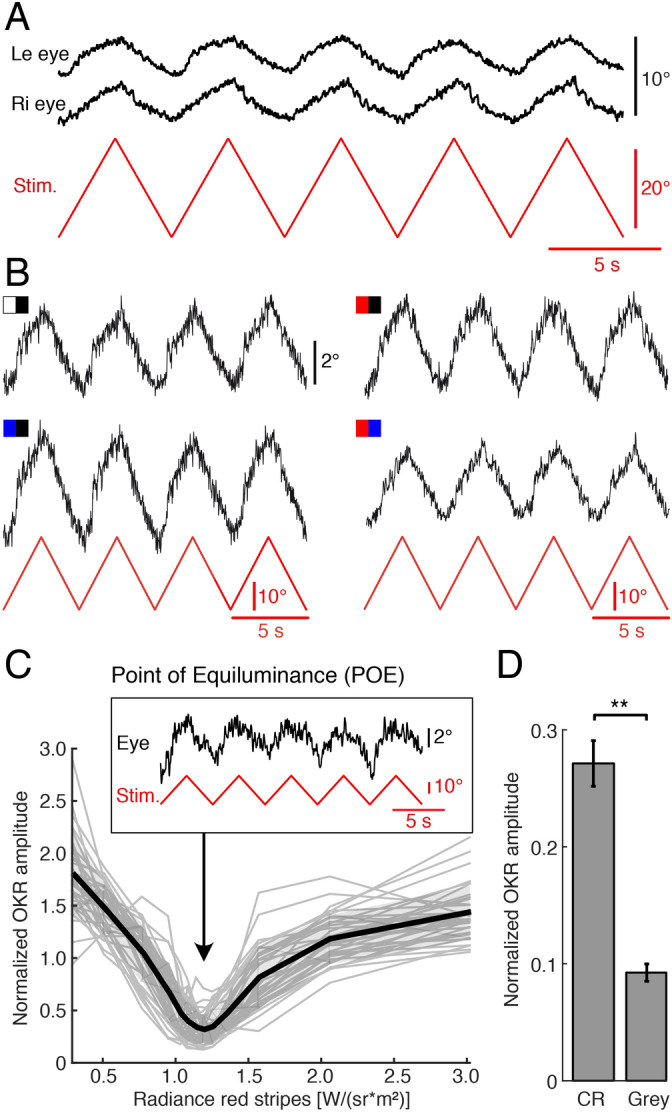
Color information contributes to large-field visual motion-induced eye movements of semi-intact preparations of *Xenopus* tadpoles. **(A)** Representative movements of the left (le) and right (ri) eye (position, black traces) evoked by alternating motion of a red-blue vertical striped pattern at constant velocity (± 10°/s; red trace). **(B)** Typical examples of eye movements in a given preparation (position, black traces) evoked by alternating motion of a black–white, black–blue, black–red and red–blue vertical striped pattern (see color-code) at constant velocity (± 10°/s; red traces). **(C)** Individual (grey traces) and averaged (black trace; *n* = 34) normalized OKR amplitudes evoked by a red-blue vertical striped motion pattern as function of the radiance of the red stripes; the inset illustrates a typical example of residual eye movements at the POE. **(D)** Bar plot depicting the response amplitude evoked by chromatic stimuli (CR) at the POE, determined individually for each animal, and control baseline amplitude (Grey). **, *p* < 0.01 (paired t-test)*.* Figure assembled with Affinity Designer (version 1.8.3).

Large-field visual motion stimulation with black/white-striped image patterns (Fig. 1A1) triggers robust conjugate ocular motor following responses of both eyes in semi-intact preparations of *Xenopus laevis* tadpoles (Fig. 2A)^10^. Horizontally alternating constant-velocity motion stimulation with a black/red- or black/blue-striped pattern (Fig. 2B) elicited eye movements with comparable magnitudes in such preparations. The robustness of these responses allowed determining the POE between the two colors (Figs. 1C, 2C). The POE at which the radiance contrast of the red stripes with respect to blue stripes with a radiance of 4.66 W*sr^−1^ m^−2^ vanished was very similar across animals and occurred on average at a value of 1.22 ± 0.09 W*sr^−1^ m^−2^ (Fig. 2C), confirming previous findings that the relative sensitivities to these two component colors show little variance between animals^12^. The normalized amplitude of the OKR at the respective individual POE, separately determined for each animal was 0.27 ± 0.15 with respect to the mean amplitude over all conditions (Fig. 2D). This residual eye movement magnitude, albeit small, was significantly greater than baseline oscillations (Two-sampled t-test: *t(42)* = 3.02, *p* = 0.004, *d* = 0.86; Fig. 2D). As a novel finding, this indicated that color contrast is sufficient to detect motion of the visual scene and to consequently evoke an ocular motor response.

### Interaction of color and radiance during OKR performance

**Figure 3:**
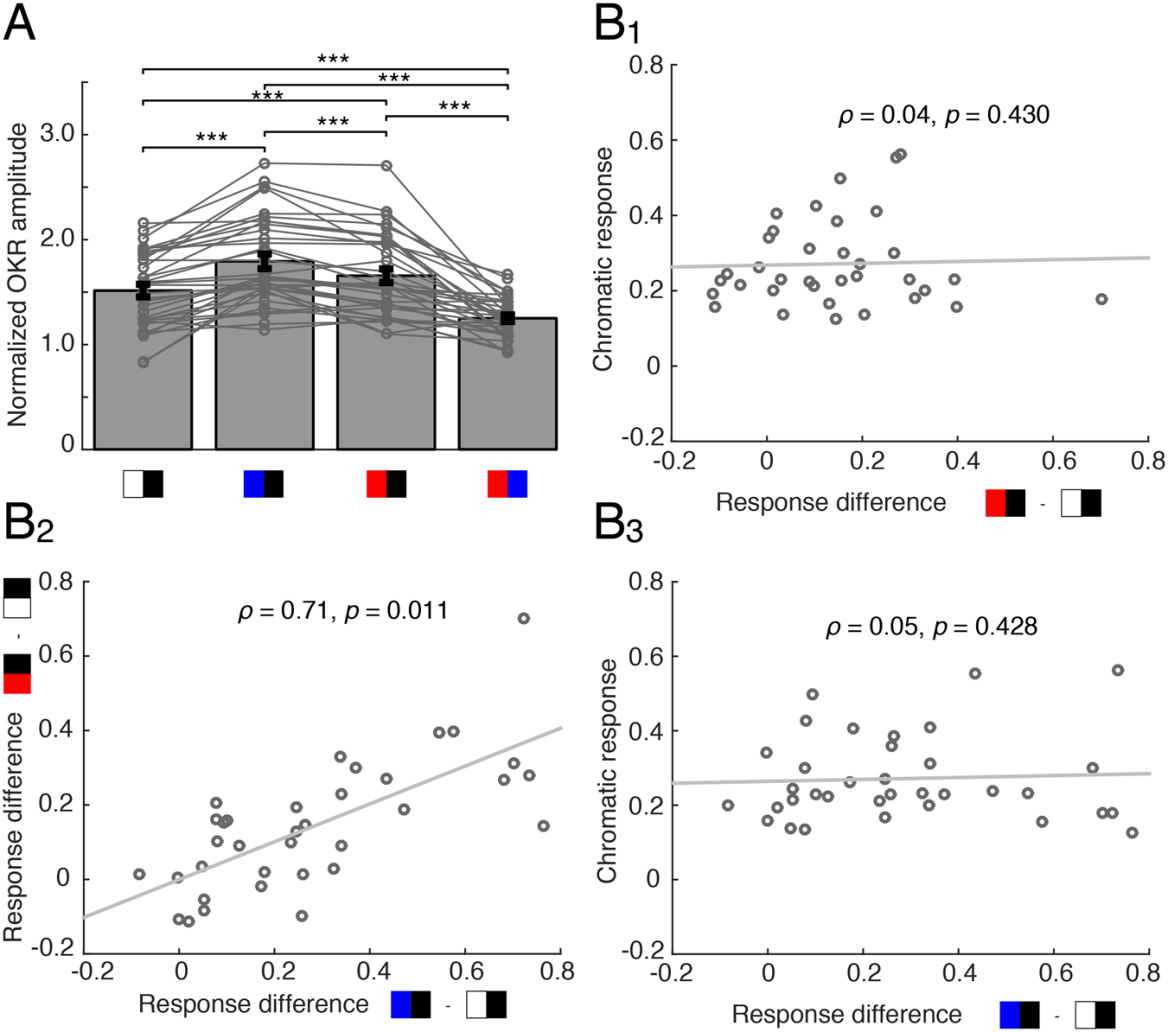
Color-dependent differential OKR performance of semi-intact preparations of *Xenopus* tadpoles. **(A)** Bar plot depicting the relative OKR amplitude for large-field visual motion stimuli with different color combinations; note the larger responses when black stripes were paired with blue or red compared to black–white stripes; ***, *p* < 0.0001 (*n* = 34; Mann–Whitney *U*-test). **(B)** Scatter plot depicting relative chromatic response (OKR amplitude at POE; see “Methods”) magnitudes (*n* = 34 animals) as function of the difference in amplitude between the conditions: red–black and white–black **(B**_**1**_**)** and blue–black and white–black **(B**_**3**_**)**; **(B**_**2**_**)** illustrates the difference in response magnitudes between blue–black and white–black (x-axis) and red–black and white–black (y-axis); despite the mathematical coupling, there is a significant correlation between blue/white and red/white response magnitudes (expected correlation due to coupling: *ρ* = 0.48). Figure assembled with Affinity Designer (version 1.8.3).

In addition to the OKR evoked by pure color contrast stimuli, high intensity color/black-striped motion stimuli provoked eye movements with amplitudes that were considerably larger compared to those elicited by a white/black-striped pattern. This is indicated by a significant main effect of stimulus color (Repeated measures ANOVA: *F* (3, 102) = 46.62, *p* < 0.001, *η*^*2*^ = 0.26; Fig. 3A) and successive post-hoc tests. This finding was rather surprising since the radiance of white stripes was approximately four-fold compared to that of the blue and more than five-fold compared to that of red the stripes (18.7 W*sr^−1^ m^−2^ compared to 4.66 W*sr^−1^ m^−2^ or 3.39 W*sr^−1^ m^−2^, respectively). In contrast, the black stripes had the same intensity. The effect persisted even when the radiance of white and colored stripes was matched (Two-sample t-test for blue: *t(5)* = − 2.90, *p* = 0.034, *d* = − 0.377). The additional color-related components, however, were not significantly correlated with the isolated chromatic OKR response at the POE (blue: *ρ* = 0.04, *p* = 0.430; red: *ρ* = 0.05, *p* = 0.428; Fig. 3B1,3). Bayes factor analysis^34^ showed, however, that there is only anecdotal evidence for a lack of effect of response difference on chromatic response (B_01_ = 2.98 for red–black minus white–black; B_01_ = 2.95 for red–black minus blue–black). The fact that additional color-related components of red and blue motion stimuli were strongly correlated (*ρ* = 0.71, bootstrap *p* < 0.011; Fig. 3B2), suggested a particular color motion sensitivity of some but not all preparations.

Furthermore, comparison between color motion-stimuli revealed significantly larger OKR amplitudes in response to a blue/black-, than to a red/black-striped motion stimulus (Figs. 2B, 3A), indicating that the performance of the OKR in *Xenopus* tadpoles differs for motion stimuli with different color patterns. Variation of pure radiance contrast of achromatic stimuli as previously employed^10^ indicated that beyond a certain contrast level, OKR amplitudes saturate. Based on these previous findings, the high contrast stimuli described above fall into the saturation range and no local sensitivity to changes in contrast is expected. Nevertheless, the increase in OKR amplitude to color stimulus motion at distinctly lower radiance contrasts compared to black/white stimuli suggests the presence of a color-related component that influences the OKR performance.

### Optic nerve population responses to colored visual motion stimuli

**Figure 4:**
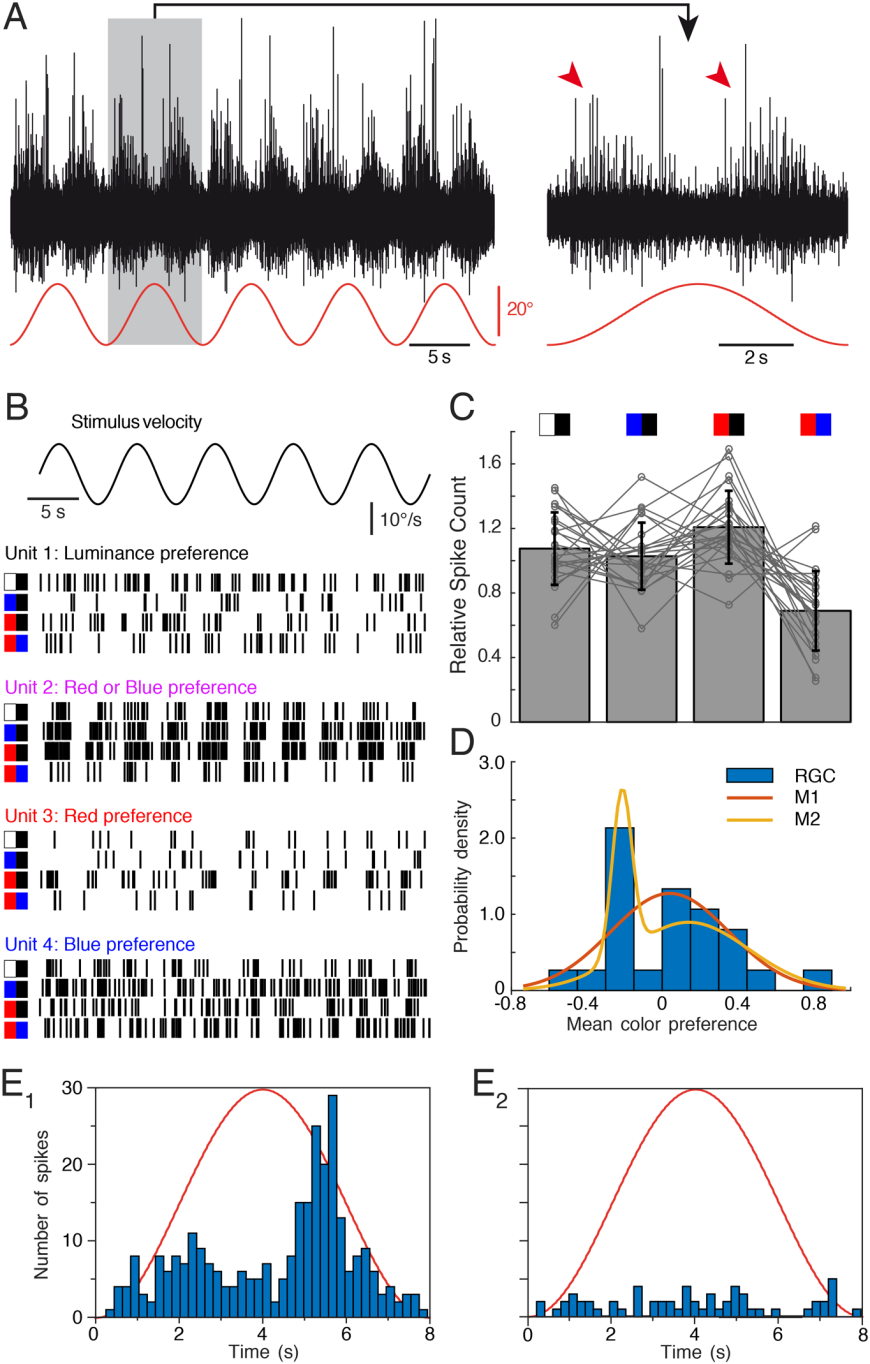
Retinal ganglion cell spike discharge during visual image motion stimulation with different color combinations in isolated optic nerves—eyes of *Xenopus* tadpoles. **(A)** Representative multi-unit spike discharge of the optic nerve in response to large-field visual motion stimulation with a black–white striped vertical pattern (red trace); the trace on the right is a magnified view of a single motion cycle (grey area) of the trace on the left; note the bidirectional velocity sensitivity (red arrow heads). **(B)** Raster plot of spike trains of four isolated individual units with different radiance or color preferences; units respond preferentially to high radiance contrast (unit 1), to blue or red versus black (unit 2) or to red or blue stimulus combinations (units 3 and 4). **(C)** Bar plot depicting relative spike counts of all isolated units (*n* = 25) during visual motion stimulation with different color combinations (icons on top). **(D)** Histogram of color preferences of *n* = 25 individual retinal ganglion cell units (RGC, blue). Negative color preference indicates preferential responses to achromatic stimuli, positive values indicate larger responses to single-colored visual motion. Red and yellow curves depict distributions from Gaussian mixture models with one (red, M1) or two (yellow, M2) components fitted to the data. **(E)** Peri-stimulus time histogram of the average spike discharge (200 ms bin width) over a single image motion cycle at 0.125 Hz (8 s) of two single units **(E**_**1**_**,E**_**2**_**)** at the POE; note the residual directional response of the unit in **(E**_**1**_**)**; red sinusoids represent stimulus cycles. Figure assembled with Affinity Designer (version 1.8.3).

Electrophysiological recordings of the optic nerve with suction electrodes consistently evoked a multi-unit discharge of retinal ganglion cells albeit with bidirectional activity patterns during horizontal stimulus motion (Fig. 4A and magnified response over one cycle). An earlier study demonstrated for radiance motion stimuli a close association between the population activity of the optic nerve and the speed of the large-field visual image motion^10^. This indicates that RGC population activity can be interpreted as surround velocity estimate. Whether this also applies when comparing responses to stimuli with distinct wavelength components—that differentially excite retinal photoreceptors—was evaluated by a set of experiments with the same color combination stimuli as used for evaluating the performance of the OKR.

Interestingly, at the population level, the relative neuronal response magnitude of the multi-unit optic nerve discharge (spike count) differed from the behavioral observations (two-sample Kolmogorov–Smirnov test, p < 0.001; compare Fig. 3A with Fig. 4C). Although the neuronal response to a high intensity red/blue-striped pattern was smaller than the response to the three other patterns (likely due to a smaller radiance contrast in this condition; Fig. 4C), the respective response magnitudes showed a different activation pattern compared to the OKR. This suggests that the mechanism that causes different optokinetic response amplitudes for different colors at high light intensities^12^ differs from the mechanism responsible for adjusting the optokinetic response simply based on radiance contrast.

### Optic nerve single-unit responses to colored visual motion stimuli

**Figure 5:**
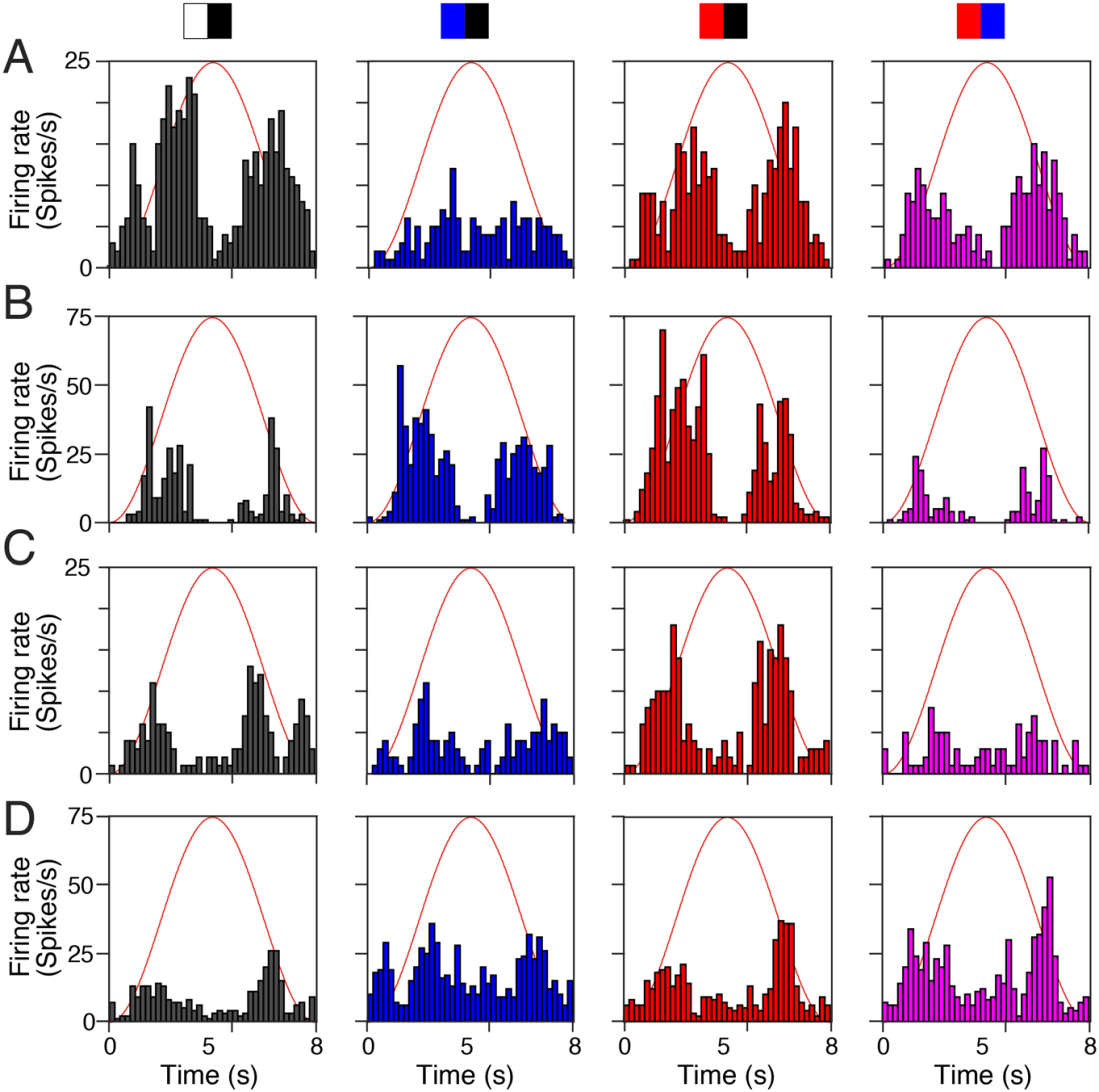
Peri-stimulus time histogram of retinal ganglion cell activity in isolated optic nerves—eyes of *Xenopus* tadpoles. **(A–D)** Peri-stimulus time histogram of the average spike discharge (200 ms bin width) over a single image motion cycle at 0.125 Hz (8 s) of the four units with different radiance or color preferences depicted in Fig. 4B as units 1–4; units respond preferentially to high radiance contrast **(A)**, to blue or red versus black **(B)** or to red or blue stimulus combinations **(C,D)**; black/white and color stripe combinations are indicated by the icons above the top row; red sinusoids represent stimulus cycles. Figure assembled with Affinity Designer (version 1.8.3).

Spike sorting of the multi-unit optic nerve discharge and isolation of individual units using SVD-based spike sorting (MATLAB R2017a) revealed an alternative mechanism for the *partially* color-specific optokinetic response amplitude (see units 1–4 in Fig. 4B and corresponding PSTHs in Fig. 5). In fact, the presence of separate subgroups of retinal motion detectors, which respond preferentially to particular wavelengths could be the origin of this color dependency. Mediation of visual motion signals in distinct color channels from the retina to the pretectum and differential coupling probability or strength within the OKR circuitry could explain the observed differential color sensitivity of this reflex.

To test this hypothesis, responses of isolated single optic nerve units (*n* = 25) that modulated their firing rate with stimulus velocity were analyzed. The color preference was identified by evaluating the response magnitude to the four high intensity color stimuli (Fig. 4C). While the total spike count varied strongly between individual units, clear differences were encountered between the color-preference of the different units, with color preferences ranging between − 0.42 and + 0.59 (10th and 90th percentile, respectively), corresponding to relative responses to colored stimuli between 66 and 181% of the same unit’s responses to white stripes. There was a roughly even split between units responding preferentially to either white (*n* = 12), or colored (red: *n* = 10, blue: *n* = 3) stripes. To support the finding that units can be separated into subtypes with preferential sensitivity to broad and narrow light spectra, respectively, Gaussian mixture models with one and two components were fitted to the units’ color preference values. Despite the additional degrees of freedom, the two-component model provided higher explanatory value, indicated by a lower Akaike Information Criterion (14.6 versus 16.9; Fig. 4D). The distinction between units with color-sensitive and color-insensitive response characteristics was obvious and allowed clear identification of a neuronal population preferentially responding to achromatic stimuli. Among color-sensitive units, however, there was no clear distinction based on the response preference to either red or blue color. In contrast to behavioral findings that animals with a strong preference for one color (expressed by their relative OKR amplitude) also show a strong response to the other color, there was no significant correlation between red and blue response preference on the level of RGC axons (*ρ* = 0.67, *p* = 0.12). Nevertheless, there was a considerable degree of variability in preference for colored or white stimuli (Fig. 4D). While most of the measured units were inactive during stimulation near the POE (Fig. 4E2), a small number of single units still clearly showed an activity profile that modulated with instantaneous stimulus velocity (see Fig. 4E1 for an example), suggesting that a set of RGC axons encode and transmit color-dependent visual motion signals.

## Discussion

The optokinetic reflex of *Xenopus laevis* tadpoles is systematically influenced by the color component of the moving visual scene. Even though eye movement amplitudes decrease with smaller radiance contrast, a residual optokinetic response at the point of equiluminance remains, even when accounting for inter-individual variance in the exact location of the POE. This color contrast-dependency complies with an activation of larger OKR amplitudes when black stripes were paired with colored instead of white stripes. The underlying neuronal computation is likely of retinal origin since single optic nerve fibers can show considerable preferences for either colored or white motion stimuli.

The residual response at the POE demonstrated that pure color contrast is a likely contributor to the visual motion detection mechanism, which forms the basis of the optokinetic reflex^10^. In fact, the OKR at POE along with the residual directionally selective response of RGCs (see Fig. 4E1) suggests that retinal motion processing contributes at least in part to the observed behavioral responses. Accordingly, by varying the radiance of the red stripes a point was reached where the behavioral response became minimal. Thus, the response at the POE for red-blue stripes indicates that the motor response is in fact elicited not just by radiance contrast, but also by color contrast. Anatomically, the necessary requirements for color vision have been shown to be present in adult *Xenopus* that possess principal and thin rods as well as four types of cones with red, blue and ultraviolet-sensitive opsins^35^. The current study provides evidence that *Xenopus* tadpoles not only possess but also recruit these anatomical structures during large-field visual motion to distinguish different colors for an adjustment of their ocular motor output. In fact, larval *Xenopus* can be trained to avoid specifically-colored segments of the environment, concluding that color vision is behaviorally relevant for these animals^36^.

Visual motion-induced behavior at the point of equiluminance facilitates a determination of the dependency of visual motion perception on color^37^. This method has been particularly applied to optomotor responses of animal models, such as flies^31^ or zebrafish^38,39^, where motion vision was found to be “color-blind”, at variance with earlier reports on amphibians such as frogs^13,14,40^ demonstrating that orienting head/body movements in both larvae and adults of *ranid* frogs are color-sensitive. If this is a fundamental difference between amphibians and zebrafish is likely but so far not yet fully explored. Although visual motion-driven ocular (this study) as well as neck/limb motor behaviors^13,14^ are color sensitive in amphibians, the color-sensitivity of the OKR, its short-latency neuronal circuitry, approximately linear input–output relationship along with the spatial specificity and the limited degrees of freedom of eye movements^41^ makes this ocular motor behavior a very sensitive assay. This is further facilitated by the possibility to isolate small responses based on their frequency characteristics and to robustly distinguish evoked reflexive from spontaneous motor activity.

Our experiments revealed a non-zero optokinetic response at the POE in the majority of preparations (26 out of 35), suggesting that color information indeed plays a role in low-level motion vision, even though the central targets of the recorded fibers and functional implication remained so far unidentified. The contribution of color to visual motion processing appears to be independent of the overall light level, provided these RGCs feed into the ocular motor reflex circuitry, since color influences the performance of the OKR also at high light intensities, when alternating white/black, red/black and blue/black stripes were presented. Such a color contribution to neuronal motion computation, however, is not restricted to brainstem levels but is also implemented in higher-order motion vision, such as for human subjective speed estimation^15^. While the speed of an equiluminant chromatic grating is only perceived to be moving at about half the speed of a corresponding luminance grating, a motion percept is still evoked in the absence of luminance contrast^17^.

The larger responses to stimuli combining black with either blue or red stripes, as compared to white/black stripes, was rather surprising, in particular since the radiance contrast of white *versus* black stripes was the highest. While obviously all tested stimulus patterns express sufficient radiance contrast for maximal optokinetic responses, colored motion stimuli likely recruit additional pathways thereby leading to increased amplitudes of the OKR. Although direct causality is still lacking, this dependency of OKR amplitudes complies with the observed pattern in the population of optic nerve fibers^10^. This indicates that radiance-dependent variations in the performance of the OKR potentially derive from retinal signal processing.

At variance with the correlation between radiance magnitudes and optic nerve population activity^10^, variations of the color composition of visual motion stimuli were generally unrelated to the population activity of retinal ganglion cells (Fig. 4C). This suggests that the observed differences in response amplitude are not caused by radiance contrast artifacts, such as reflections. Rather, the increase of the OKR amplitude during chromatic stimulation could be explained by retinal motion detectors with preferences for specific colors, which differ in how strongly they are coupled to the brainstem OKR circuitry^39^. This hypothesis is supported by our observation of individual units at the optic nerve level with varying preferences for large-field visual motion stimuli in red, blue or white color (Fig. 4B). Evaluating individual units based on how their response changes to white and red or blue stimuli, respectively, showed a relatively large separation between two types (see Fig. 4C). In ~ 50% of the recorded units, the spike activity decreased when color-striped visual motion stimuli were presented, while in the remaining 50% of the examined optic nerve fibers the spike discharge rate increased. This latter augmentation was typically similar for red- and blue-striped motion stimuli, although, units with a preference for either one of the two colors were found. This demonstrates further that a neuronal substrate for color-specific processing of motion information is implemented in the *Xenopus* as in the zebrafish retina. In the present study, however, it was not possible to clarify whether the analyzed optic nerve fibers were indeed connected via the accessory optic system to the relevant pretectal relay nucleus^4^ and thus in fact have contributed to the OKR response. While most of the optic nerve fibers investigated here (examples shown in Fig. 5) did not show a directional preference that would be required to sustain the OKR response (for an exception see Fig. 4E1), their response to both color and motion indicates that color-sensitive motion channels already exist in the amphibian retina and may constitute the origin of color-sensitive OKR responses compatible with the color-sensitivity of optomotor reactions of the head/body^40^.

From an ecological point of view, color information is advantageous for OKR performance. Color provides a number of additional cues about the environment and the visual motion within it and potentially extends the sensitivity of this motor behavior. Color increases the saliency of objects, and thus facilitates motion perception^19^. In addition, certain environmental features have invariant colors and—based thereupon—can be more easily distinguished from other objects. This plays a particular role in the distinction between visual motion of the entire scene (which is most likely caused by self-motion and therefore should elicit an optokinetic ocular motor response), and motion of external objects such as floating debris in aquatic environments for instance. One particular example in this environment is a distinction between the blue sky, which always provides a world-stationary reference, in contrast to other environmental features which might be moving themselves, such as the surrounding fauna. Color cues can thus assist in performing a behavioral distinction between different sources of visual motion, in particular self- and object motion.

## Data Availability

The datasets generated and analyzed during the current study are available from the corresponding author on reasonable request.
